# Editorial: Advances in Ascochyta Research, Volume II

**DOI:** 10.3389/fpls.2023.1290189

**Published:** 2023-09-19

**Authors:** Jennifer Davidson, Weidong Chen, Diego Rubiales, Yongle Li

**Affiliations:** ^1^ South Australian Research and Development Institute (SARDI), Adelaide, SA, Australia; ^2^ United States Department of Agriculture - Agricultural Research Service (USDA-ARS), Washington State University, Pullman, WA, United States; ^3^ Institute for Sustainable Agriculture, Spanish National Research Council (CSIC), Cordoba, Spain; ^4^ Australian Grain Technologies, Roseworthy, SA, Australia

**Keywords:** ascochyta blight, lentil (*Lens culinaris*), faba bean (*Viciae faba*), chickpea (*Cicer arietenum*), field pea (*Pisum sativum*)

This Research Topic is the second of the Advances in Ascochyta Research series. Legume crops provide an excellent source of high quality plant protein and have a key role in arable crop rotations reducing the need for fertilizer application and acting as break-crops. However, these crops are affected by a number of foliar and root diseases, with ascochyta blights being among the most important group of diseases worldwide. Ascochyta blights are incited by different pathogens in the various legumes. A number of control strategies have been developed including resistance breeding, cultural practices and chemical control. However, only marginal successes have been achieved in most instances, most control methods being uneconomical, hard to achieve or resulting in incomplete protection. There is a need for a co-operative research on these diseases, from agronomy to breeding, covering traditional and modern genomic methodologies.

The ascochyta pathogens are spread by both asexually and sexually produced spores, the latter derived from two mating types, denoted MAT1-1 and MAT1-2, on faba bean (*Ascochyta fabae/Vicia faba*), lentil (*Ascochyta lentis*/*Lens culinaris*) and chickpea (*Ascochyta rabiei*/*Cicer arietinum*). Understanding the role of these mating types can assist in determining risk of epidemics and the need for control strategies. In glasshouse studies, the MAT1-2 mating type of *Ascochyta fabae* was found to be more pathogenic than MAT1-1 on faba beans in Tunisia (Youssef et al.) suggesting that where this mating type was prevalent, the epidemic risk was greatest. The two mating types co-existed across all regions of the surveyed area but an imbalance of MAT1-2 occurred in some regions, signifying a geographical variation in disease risk. A wide distribution of the two mating types is likely to lead to changes in virulence genes in the pathogen populations and subsequent loss of resistance in host cultivars. Studies in southern Australia (Blake et al.) on *A. fabae* found an equal distribution of the two mating types across the growing region. A host set was inoculated with 154 isolates of *A. fabae* and 80% of the population were identified as belonging to a single pathogenic group, able to infect widely grown commercial cultivars. Prior to 2015 only a few isolates were able to infect this host set, reflecting the spread of the aggressive form of this pathogen in a few years. Another emerging pathogenic group was also identified that could infect elite cultivars with a high level of resistance. These studies underline the need for continual monitoring of pathogen populations to determine the epidemic risks and inform breeding programs on effective resistances.

The genetic resistance to *A. rabiei* in chickpea is complex and much is still unknown. GWAS (genome-wide association) analysis of resistance to two highly aggressive single spore isolates identified twenty-six genomic regions on Ca1, Ca4, and Ca6 that showed significant association with resistance to ascochyta blight (Raman et al.). Eighty-nine significantly associated single nucleotide polymorphisms (SNPs) were located within candidate genes, including genes encoding for serine/threonine-protein kinase, Myb protein, quinone oxidoreductase, and calmodulin-binding protein all of which are implicated in disease resistance. Carmona et al. used recombinant inbred lines (RIL) to identify SNPs in four genomic regions associated with ascochyta blight resistance in chromosomes 2 and 4. A total of 30 genes from the identified regions were selected as robust candidates, and including a leucine-rich repeat receptor-like protein kinase, as the most robust candidate gene, as it plays critical roles in plant stress responses and immunity. These studies identify valuable sources of genetic resistance, SNP markers and candidate genes underlying genomic regions associated with ascochyta blight resistance which may enable chickpea breeding programs to make genetic gains via marker-assisted/genomic selection strategies.

Breeding programs must use methods of resistance screening that reflect field conditions and often this requires a mix of controlled environment and field screening. Sharma et al. used multi-environment testing and GGE biplot analysis to identify ascochyta blight resistance genotypes of chickpea and also the ideal test locations in India suitable for ongoing host germplasm screening. Controlled environment screening was seen as a means to reduce large plant populations via initial screening to remove highly susceptible types.

Fungicide usage is an important component of controlling epidemics of ascochyta blight in legume crops but repeated applications risk the development of fungicide resistance in the pathogen populations. Fonseka et al. in North Dakota, USA, found isolates of *A. pinodes* and *A. pisi*, causal pathogens of ascochyta blight on field pea, had lower sensitivity to pyraclostrobin and prothiaconazole fungicides than baseline isolates. Disease control with these products in greenhouse conditions was significantly lower against isolates identified as having reduced sensitivity, compared to isolates with baseline sensitivity. Fungicides that combine active ingredients with different modes of action can reduce this risk. Fanning et al. investigated a range of products using preventative sprays (applied before a rainfall event) and curative action (applied at first sign of disease post-infection). Greater yield losses from disease were associated with the post-infection sprays, particularly in higher rainfall regions although in lower rainfall environments the post-infection sprays could be economic. All products, single active or multiple actives, provided some control over disease, and their effectiveness allows growers to rotate fungicides, reducing the risk of fungicide resistance.

## Author contributions

JD: Writing – original draft. WC: Writing – review & editing. DR: Writing – review & editing. YL: Writing – review & editing.

